# A Case of Idiopathic Central Diabetes Insipidus and a Mosaic Form of Turner Syndrome

**DOI:** 10.7759/cureus.71487

**Published:** 2024-10-14

**Authors:** Pavel E Stanchev, Ekaterina S Babadzhanova-Hristova, Maria M Orbetzova

**Affiliations:** 1 Department of Endocrinology and Metabolic Diseases, Medical University of Plovdiv, Plovdiv, BGR

**Keywords:** diabetes insipidus, hypergonadotropic hypogonadism, idiopathic, oligomenorrhea, turner syndrome, аntidiuretic hormone deficiency

## Abstract

Central diabetes insipidus is a clinical syndrome caused by the loss of function of vasopressinergic neurons in the hypothalamus, which results in impaired secretion of arginine vasopressin (AVP). AVP deficiency leads to the inability to concentrate urine, resulting in hypotonic polyuria and polydipsia. The condition is most often acquired, but in some cases, the etiology remains unknown, in which the disease is classified as idiopathic.

Turner syndrome is the most common sex chromosome abnormality in women, caused by complete or partial absence of one of the two X chromosomes. In some cases of Turner syndrome, an abnormal cell division occurs during the early stages of the fetal development, resulting in mosaicism: some cells in the body possess two complete copies of the X chromosome, while others have only one.

The coexistence of Turner syndrome and diabetes insipidus is extremely rare but should nevertheless be sought in all patients through focused clinical thinking and testing, as both conditions have long-term health consequences and should be promptly diagnosed and treated.

We report a clinical case of a 22-year-old female patient, diagnosed with idiopathic central diabetes insipidus and a mosaic form of Turner syndrome, presenting with polyuria and polydipsia. The performed water deprivation and desmopressin tests proved a central form of diabetes insipidus. The imaging studies that were conducted, an MRI of the hypothalamic-pituitary region in particular, revealed the existence of a "dark" type microadenoma with discrete compression of the infundibulum. The patient was started on vasopressin replacement treatment with a good therapeutic effect. In the follow-up imaging studies, the structure of the pituitary gland showed no dynamics. There are only a few cases of simultaneous development of central diabetes insipidus and Turner syndrome that have been described in the literature. Further research is needed in order to discover the connection between the pathogenesis of the development of antidiuretic hormone deficiency and Turner syndrome.

## Introduction

Diabetes insipidus is a chronic disease characterized by the presence of polyuria and polydipsia as a result of insufficient secretion or reduced effect of antidiuretic hormone at the level of the distal tubule. Diabetes insipidus is classified as central, when there is a deficiency of antidiuretic hormone, and nephrogenic, when resistance to its action is observed in the renal tubules. Diabetes insipidus is a rare disease with an incidence of about 1:25000 [1]. The condition is considered idiopathic in 20% to 50% of the affected subjects [2].

Turner syndrome, as the most common sex chromosome abnormality, affects 1:2000 to 1:2500 live female births and is diagnosed, based on clinical presentation, combined with a genotype consisting of one normal X chromosome and a complete or partial absence of the other X chromosome [3]. Female patients with 45,X/46,XY mosaic forms of Turner syndrome present with different phenotypic variations, the most common of which is the mixed gonadal dysgenesis, and other very rare cases of phenotypic male characteristics, genital ambiguity, or normal female secondary sex characteristics [4]. The management of Turner syndrome includes strict evaluation for other associated abnormalities: cardiac, renal, and autoimmune diseases. A number of patients with a mosaic form of Turner syndrome lack the typical signs, such as a wide or weblike neck, low-set ears, and a broad chest with widely spaced nipples, which can be a reason for a late diagnosis. The coexistence of Turner syndrome and diabetes insipidus is rare, but it is important to highlight the need for a targeted clinical investigation in order to find symptoms of polyuria, nicturia, and polydipsia in those patients, as the condition could be life-threatening if there is an inability to compensate the water losses during dehydration. Severely reduced or absent ovarian reserve and hypergonadotropic hypogonadism in patients with a mosaic form of Turner syndrome should receive timely specialized help for cryopreservation of ovarian tissue or oocytes.

We present a rare clinical case of a 22-year-old female patient with idiopathic central diabetes insipidus and a mosaic form of Turner syndrome.

## Case presentation

A 22-year-old female patient was admitted to the Clinic of Endocrinology at University Hospital “St. George” due to increased fluid intake (8-9 liters per day) for about a month, as well as frequent urination, including nicturia (3-4 times at night). She reported disturbances in the menstrual cycle, manifesting with oligomenorrhea, with menarche at 12 years of age, without any pregnancies. She had an older sister with primary amenorrhea. No accompanying diseases or systemic medication intake were noticed.

Physical examination showed no skin abnormalities, with no evidence of hirsutism, acanthosis, or galactorrhea. The habitus was normosthenic. The height of the patient was 168 cm, the body weight was 52 kg, and the calculated BMI was 18.4 kg/m2. Vesicular breathing bilaterally, without wheezing. Rhythmic heart activity with a frequency of 78-80 beats/min and an arterial pressure of 110/60 mmHg. No heart murmurs were detected by auscultation. The abdomen is soft and painless with normal physiological peristalsis, the liver and spleen are not palpable, and there are no signs of edema in the limbs.

Blood tests were performed (Table 1) and showed normal blood glucose levels and electrolytes and preserved renal function. Hormonal results showed no evidence of manifest hypopituitarism.

**Table 1:** Basic investigations of the patient TSH: thyroid-stimulating hormone, FT4: free thyroxine, FT3: free triiodothyronine, LH: luteinizing hormone, FSH: follicle-stimulating hormone, ACTH: adrenocorticotropic hormone

Lab test	Value	Reference range
Fasting glucose	4.41	2.8-6.1 mmol/l
Serum calcium	2.45	2.12-2.62 mmol/l
Serum potassium	5.3	3.5-5.6 mmol/l
Serum sodium	145	136-151 mmol/l
Urea	3.1	2.6-7.2 mmol/l
Creatinine	73	44-96 µmol/l
TSH	1.662	0.34-5.6 mU/L
FT4	10.1	7.86-14.41 pmol/l
FT3	4.47	3.8-6.0 pmol/l
Prolactin	363.13	70-565 mU/L
LH	3.58	2.12-10.89 IU/L
FSH	8.14	3.85-8.78 mIU/ml
Estradiol	107.99	24-114 pg/ml
Cortisol – 8 h	304.45	154-624 nmol/l
Cortisol – 23 h	40.27	nmol/l
ACTH	31.65	5-47 pg/ml

After performing the water deprivation test (Table 2), the results were analyzed, and the diagnosis of diabetes insipidus was established. The patient underwent a therapeutic test with desmopressin acetate 2x0.1 mg/daily. After the initiation of the medication, the patient experienced an improvement in her general condition with a reduction of polydipsia, polyuria, and nicturia. Central diabetes insipidus was observed, and an MRI of the hypothalamic-pituitary region was scheduled.

**Table 2:** Water deprivation test

Time	Weight	Urine volume	Serum osmolality	Urine osmolality
08:00	52.3	225	308.11	74
09:00	52.1	220	-	-
10:00	51.9	208	310.3	78
11:00	51.7	200	-	-
12:00	51.5	195	314.5	86

**Figure 1:**
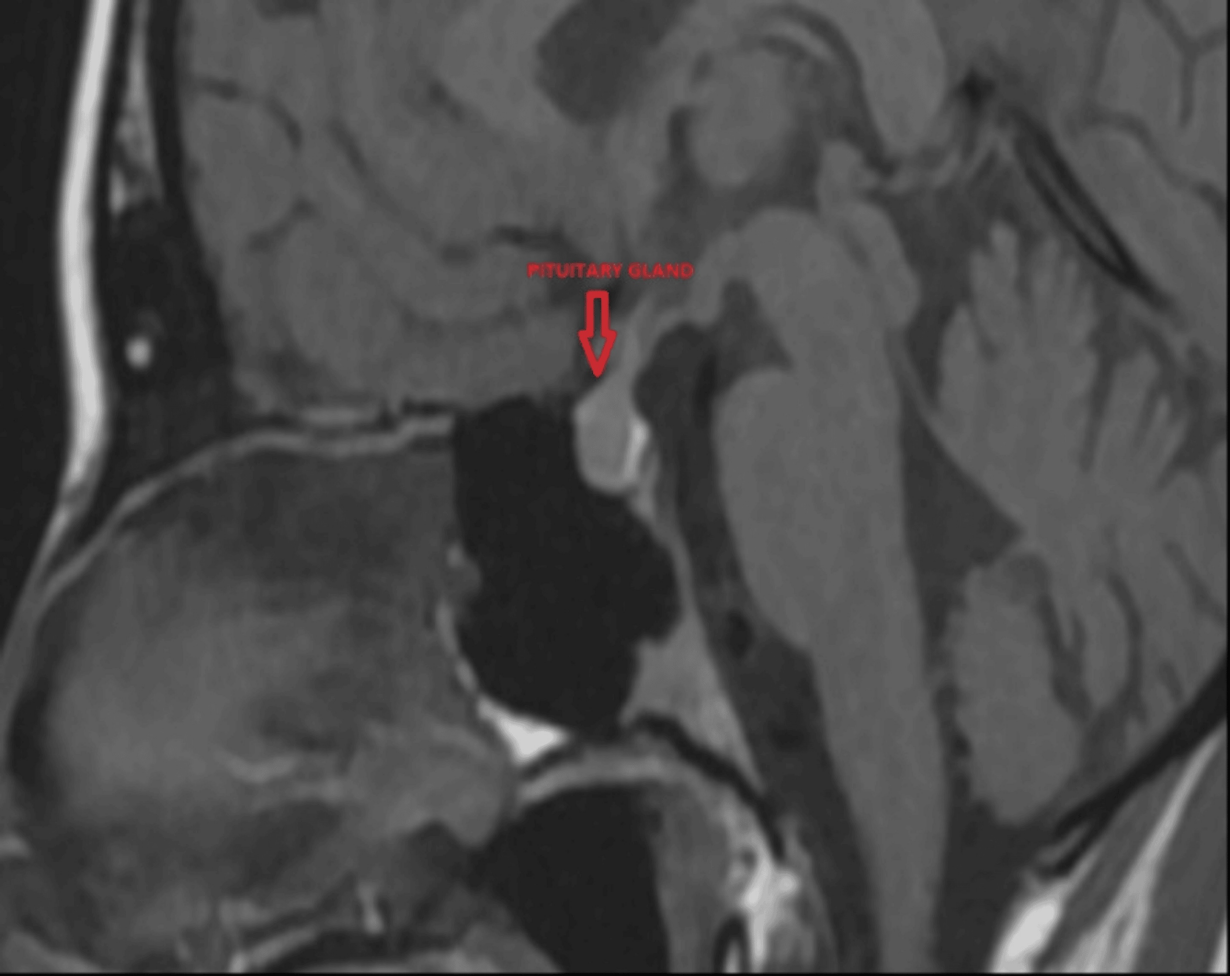
MRI of the pituitary gland - native series, sagittal plane Pituitary microadenoma type "dark" with discrete compression of the infundibulum. MRI: magnetic resonance imaging

An MRI of the brain was performed (Figure 1). The pituitary gland presented in upper border dimensions, with a pronounced central concavity along the upper contour and a strongly shortened infundibulum. The intrasellar structures had a normal position with a good differentiation between the neuro- and adenohypophysis. Inhomogeneity in the structure of the pituitary was observed. Medially, sub- and post-infundibularly, a hypointense, small zone without a defined shape and size was visualized, which after the application of the contrast material formed a hypointense nodule with a diameter of 4.6 mm, causing compression of the infundibulum ventrally: picoadenoma. No exteriorization of the process was found. The para- and suprasellar anatomical complex was normal. The ventricular system was centrally located with preserved capacity. The conclusion was that MRI data of a pituitary picoadenoma of type "dark" was present.

The visualized microadenoma, causing compression of the infundibulum, was discussed as a possible etiologic cause of the central diabetes insipidus. A consultation with a neurosurgeon was performed, and transsphenoidal adenomectomy was recommended. Unfortunately, the patient did not give her consent. Given the absence of manifest hypopituitarism, the young age of the patient, and the lack of a definite relationship between the MRI finding and the presence of central diabetes insipidus, the medical team strictly followed up on the patient, having in mind the possible dynamics in the size of the pituitary microadenoma as well as the appearance of hormonal disturbances.

About half a year after the establishment of the diagnosis and the initiation of desmopressin substitutional therapy, the patient presented with oligomenorrhea. Studies of gonadotropic hormones and estradiol in the follicular phase of the menstrual cycle have been conducted several times (Table 3).

**Table 3:** Hormonal results during the follow-up obtained in the follicular phase of the menstrual cycle LH: luteinizing hormone, FSH: follicle-stimulating hormone

Test	First month	9 months	10 months	22 months	23 months	Reference
LH (follicular phase)	3.58	86.38↑	34.89↑	17.38↑	6.53	2.12-10.89 IU/L
FSH (follicular phase)	8.14	131.14↑	49.13↑	34.24↑	5.85	3.85-8.78 mIU/ml
Estradiol (follicular phase)	107.99	39.31	148.45	70.22	265.12	24-114 pg/ml

A constellation of hypergonadotropic hypogonadism was established, a consultation with a gynecologist was performed, and rudimentary ovaries and condylomatosis of the labia majora were observed. Because of the family history of primary amenorrhea in the patient's sister, a genetic cause of the present menstrual disturbances, ovarian changes, and hypergonadotropic hypogonadism was discussed.

FISH analysis of a DNA probe was performed, analyzing 185 interphase nuclei. In 97.3% of the analyzed nuclei, two green signals for the chromosomal locus (Xp 11.1 - q11.1) of the X chromosome - alfa satellite (DXZ1) green were detected. One green signal was detected in 2.7% of the analyzed nuclei. From the consultation with a geneticist and the results of the analyses, a low-grade mosaic of Turner syndrome was observed.

**Figure 2:**
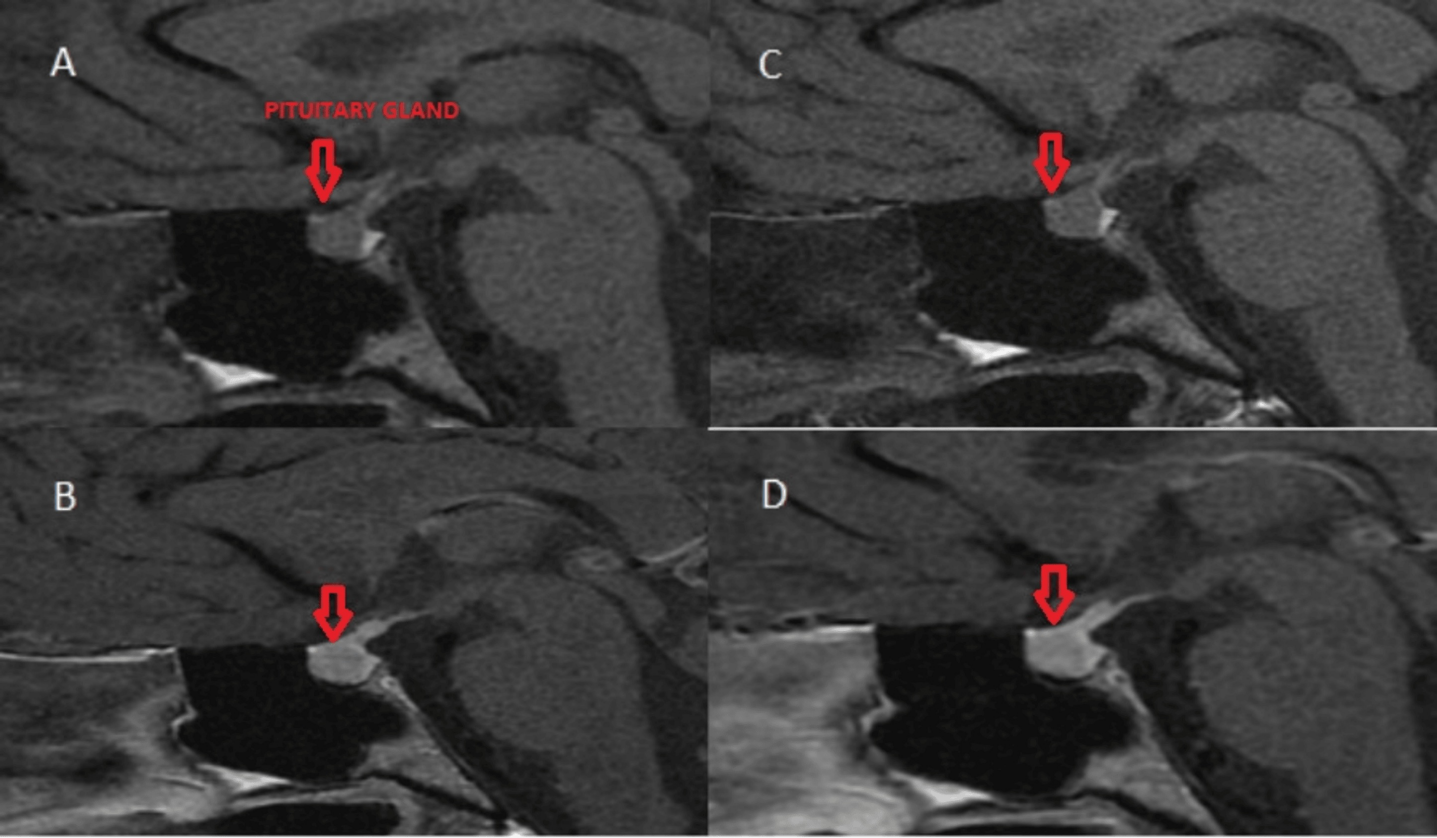
Control MRI of the hypothalamic-pituitary region in the sagittal plane shows no changes in the findings A: native series, B: contrast series (22 months after the first MRI), C: native series, D: contrast series (31 months after the first MRI) MRI: magnetic resonance imaging

A follow-up MRI with contrast material of the pituitary gland was performed 22 months (Figure 2A-2B) and 31 months after the diagnosis (Figure 2C-2D). No dynamics were noted.

## Discussion

Central diabetes insipidus occurs equally in both sexes and is a condition in which the synthesis, release, and transport of antidiuretic hormones are impaired with the subsequent manifestation of polydipso-polyuric syndrome [5]. In most patients, the disease is due to the destruction and degeneration of the neurons, originating in the paraventricular and supraoptic nuclei. Etiological causes of central diabetes insipidus could be craniopharyngioma, germ cell tumors, vascular diseases, local autoimmune and inflammatory diseases, sarcoidosis, histiocytosis, trauma, metastases, surgical interventions, and cranial malformations [6].

In the described clinical case of central diabetes insipidus, serial MRI scans were performed with visualization of a microadenoma, without dynamics in the size during the follow-up. Probably, the described discrete compression of the infundibulum has no etiological significance for the occurrence of the disease. The diagnosed central diabetes insipidus is probably of idiopathic origin. The visualized compression of the infundibulum does not cause hypopituitarism, as evidenced by the subsequently available data on hypergonadotropic hypogonadism. The occurrence of menstrual disorders and the established hypergonadotropic hypogonadism, combined with the history of primary amenorrhea in the patient's sister, points to a genetic cause of the patient's menstrual disturbances. The performed FISH analysis proved the presence of a low-grade mosaic form of Turner syndrome. The patient did not possess any phenotypic characteristics of the classic genetic abnormality.

In Turner syndrome, in addition to the characteristic phenotypic marks and abnormalities of the internal organs, a number of endocrine disorders may occur: growth disorders (95-100%), hypergonadotropic hypogonadism (90-95%), prediabetes (15-50%), diabetes mellitus type 2 (10%), and thyroiditis and hypothyroidism (15-30%) [7].

To our knowledge, very few clinical cases of the coexistence of Turner syndrome and central diabetes insipidus have been described in the literature. Kang et al. described idiopathic central diabetes insipidus in a 12-year-old girl, diagnosed with Turner syndrome due to short stature, obesity, hyperlipemia, and diabetes mellitus. Because of the appearance of polydipsia and polyuria, a water deprivation test and an MRI of the pituitary gland were performed, showing a thickening of the infundibulum of the pituitary gland. Desmopressin treatment was started [8]. Balkin et al. also described a patient with Turner syndrome and idiopathic central diabetes insipidus back in 1978 [9].

The described clinical case is unique because a mosaic form of Turner syndrome was found in a patient diagnosed with central diabetes insipidus with a probable idiopathic etiology. A possible link between genetic pathology and pituitary disease has been suggested, and future in-depth research is needed to prove it. The patient was informed about the high future risk of reproductive disorders that arises from the existing mosaic genetic anomaly.

## Conclusions

We present a very rare clinical case of central diabetes insipidus in a patient with a mosaic form of Turner syndrome and a normal phenotype in order to emphasize the need for continued, structured, and multidisciplinary monitoring and screening of patients with Turner syndrome, thus facilitating the early diagnosis of the existing comorbidities. It is important to recognize the development of central diabetes insipidus of autoimmune or idiopathic etiology, as appropriate and timely therapy could prevent several health complications and also help to extend the life of these patients. The diagnostic process in patients with central diabetes insipidus should not be put to an end, as it is possible for some of them to have mosaic forms of genetic disorders that should be further investigated. The relationship between the pathological conditions presented is a field for further future research.
